# Insect-based models in pharmaceutical ecotoxicology: a bibliometric and narrative review

**DOI:** 10.1080/19336934.2026.2692827

**Published:** 2026-06-30

**Authors:** Jumriani Jumriani, Ratnawati Ratnawati, Youdiil Ophinni, Emil Salim, Firzan Nainu

**Affiliations:** a Faculty of Pharmacy, Hasanuddin University, Makassar, Indonesia; b The Hakubi Center for Advanced Research, Kyoto University, Kyoto, Japan; c Center for Southeast Asian Studies, Kyoto University, Kyoto, Japan; d Immunology Frontier Research Center, Osaka University, Suita, Japan; e Center for Infectious Diseases, Kobe University, Kobe, Japan; f Department of Pharmacology and Clinical/Community Pharmacy, Faculty of Pharmacy, Universitas Sumatera Utara, Medan, Indonesia; g Department of Pharmacy, Faculty of Pharmacy, Hasanuddin University, Makassar, Indonesia; h Unhas Fly Research Group, Faculty of Pharmacy, Hasanuddin University, Makassar, Indonesia

**Keywords:** Pharmaceutical waste, ecotoxicology, trophic transfer, insects, *Hermetia illucens*, *Drosophila melanogaster*

## Abstract

Pharmaceutical waste has been recognized as an emerging contaminant that is increasingly detected in the environment and may pose ecotoxicological risks. This review aims to describe the global development of research on pharmaceutical waste within the field of ecotoxicology and to examine the role of insects in trophic food-chain exposure pathways. A bibliometric analysis was conducted using the Scopus database, comprising 1,051 articles published between 2010 and 2025. The results show a consistent upward trend in publications, with pharmaceutical ecotoxicology research predominantly focused on aquatic environments, environmental risk assessment, contaminant monitoring, and toxicological evaluation. Keyword analysis further indicates that terrestrial exposure pathways remain comparatively less emphasized within the current bibliometric landscape. The narrative review indicates that pharmaceutical waste can accumulate in organisms and be transferred through the food chain, leading to various physiological and systemic toxic effects, even at low environmental concentrations. In this context, insects play a strategic role as mediators of trophic exposure. *Hermetia illucens* is relevant in waste management and biotransformation processes, while *Drosophila melanogaster* is a well-established model organism for investigating physiological and molecular responses to pharmaceutical compound exposure. Thus, this review discusses the potential of an insect-based ecotoxicology framework in which *H. illucens* may serve as a bioconversion model for waste processing and *D. melanogaster* as a mechanistic model for toxicological studies. We also highlight the need for a more integrative ecotoxicological approach to pharmaceutical waste that explicitly considers insect involvement and food chain – based exposure pathways.

## Introduction

Pharmaceutical waste has been identified as an emerging contaminant that is receiving increasing attention in the field of ecotoxicology [1–3]. This waste is generated across the entire pharmaceutical lifecycle, from industrial manufacturing and healthcare delivery to veterinary practice, agricultural use, and household consumption. Once released, pharmaceutical residues can enter the environment through sewage systems, soil, and surface or groundwater [4–6]. Their presence raises serious concerns because pharmaceutical compounds are specifically designed to be biologically active at low concentrations, making them capable of exerting toxic effects even on non-target organisms [6,7]. Moreover, their long-term persistence and accumulation in the environment may contribute to the development of antibiotic resistance, endocrine disruption, and the degradation of overall ecosystem quality [3,6,7].

Ecotoxicology is a branch of environmental toxicology that examines the effects of chemical contaminants on organisms and their interactions within ecosystems. To date, research in pharmaceutical ecotoxicology has predominantly focused on aquatic ecosystems, largely employing fish [8], algae, and crustaceans as test organisms [9]. This emphasis reflects the fact that aquatic systems are major sinks for pharmaceutical residues. While such an approach is highly relevant, the dominance of aquatic models has resulted in a limited understanding of pharmaceutical impacts on terrestrial organisms. In terrestrial ecotoxicology, several non-aquatic organisms, including soil invertebrates and pollinators, have also been used to evaluate the ecological impacts of environmental contaminants because of their important roles in ecosystem functioning and trophic interactions [10,11]. Insects, despite their extensive use as model organisms in biomedical research and toxicology, remain comparatively underrepresented in pharmaceutical ecotoxicology studies. Insects are ecologically relevant because they contribute to decomposition processes, trophic interactions, and contaminant transfer within terrestrial ecosystems, while also serving as experimental systems for investigating physiological and molecular responses to environmental contaminants.

In this context, insects offer considerable potential for advancing research in pharmaceutical ecotoxicology. The black soldier fly (*Hermetia illucens*) has been widely investigated as a bioconversion agent capable of processing diverse organic wastes, including those containing antibiotic residues and other pharmaceutical compounds [12–14]. Its ability to tolerate and transform contaminants makes this species a promising candidate for bioremediation-oriented studies. Meanwhile, the fruit fly (*Drosophila melanogaster*), a well-established model organism in genetics and toxicology, provides a powerful platform for evaluating the physiological, biochemical, and molecular effects of pharmaceutical exposure. These include impacts on survival, reproduction, oxidative stress, and gene expression, which are relevant endpoints for ecotoxicological assessment [15,16].

This article integrates bibliometric and narrative review approaches to provide a comprehensive overview of pharmaceutical ecotoxicology research. The bibliometric analysis maps the global development of the field, identifying publication trends, influential contributors, and existing research gaps. Subsequently, the narrative review explores the role of insects, particularly *H. illucens* and *D. melanogaster*, as potentially useful and complementary models in pharmaceutical ecotoxicology. Through this integrated approach, the article discusses how insects may contribute to pharmaceutical ecotoxicology research through potential applications in environmental bioremediation and toxicological assessment, thereby complementing conventional models used to evaluate pharmaceutical contamination.

## Methods

### 2.1. Data collection

**Figure 1.**
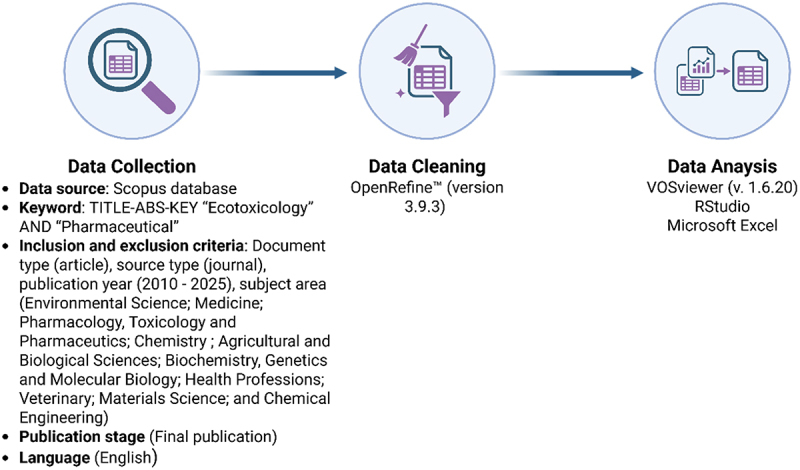
Overview of the literature search and bibliometric workflow.

This study applies bibliometric methods to review the literature related to pharmaceutical ecotoxicology (Figure 1). Several databases, including Scopus, Web of Science (WoS), and Google Scholar, are commonly used to provide bibliographic and citation records for bibliometric studies [17]. However, in the present study, Scopus was selected as the sole data source for the bibliometric analysis because of its extensive literature coverage, high-quality metadata, and compatibility with bibliometric analysis tools [18,19].

Data collection was conducted on 13 December 2025, using the search string TITLE-ABS-KEY (‘Ecotoxicology’ AND ‘Pharmaceutical’), resulting in 1,588 documents. The data were filtered to publications from 2010 to 2025, English-language documents, journal source types, final-stage publications, and article document types. Considering the broad range of scientific disciplines, data filtering involved eleven subject areas: Environmental Science; Medicine; Pharmacology, Toxicology and Pharmaceutics; Chemistry; Agricultural and Biological Sciences; Biochemistry, Genetics and Molecular Biology; Health Professions; Veterinary; Materials Science; and Chemical Engineering. The final dataset consisted of 1,051 documents.

### 2.2. Data cleaning

After the initial data collection, the Scopus database was downloaded and cleaned using OpenRefine™ (version 3.9.3) to minimize bias caused by inconsistencies and word duplication. This process aimed to improve the quality of the units of analysis by identifying spelling variations and merging items that represent the same concept. For example, *ecotoxicologia* and *ecotoxicity* were combined into *ecotoxicology*, *micropollutant* into *micropollutants*, and *waste water* into *wastewater*. The cleaned dataset was then exported in a format compatible with the bibliometric software and prepared for analysis [19,20].

### 2.3. Data analysis

The cleaned data from OpenRefine™ were then exported to three main applications. VOSviewer version 1.6.20 (Leiden University, Leiden, The Netherlands) was used to map and visualize bibliometric networks. These visualizations, often referred to as maps, enable network analyses such as co-authorship, co-occurrence, and co-citation, which can be applied to authors, publication sources, countries, and keywords. RStudio version 2025.09.2.0 (Posit Software, PBC, Boston, MA, USA) was used for additional data processing and visualization. Excel 365 (Microsoft Corporation, Redmond, WA, USA) was used to refine and customize graphical outputs [18,20–23].

### 2.4. Narrative review methodology

Literature for the narrative review was retrieved from Scopus and PubMed using keyword combinations related to pharmaceutical waste, pharmaceutical exposure, ecotoxicology, trophic transfer, bioaccumulation, *Hermetia illucens*, and *Drosophila melanogaster*. Additional relevant articles were identified through manual screening of reference lists.

Studies discussing pharmaceutical exposure, trophic transfer, toxicological responses, bioaccumulation, and insect-based model systems were included, whereas studies focused exclusively on therapeutic or clinical applications were excluded. To distinguish ecotoxicological evidence from pharmacological or biomedical studies, priority was given to investigations that used environmentally relevant exposure concentrations (i.e. within ranges reported in environmental monitoring data) or that explicitly assessed ecologically meaningful endpoints such as mortality, development, reproduction, behaviour, or trophic transfer. Studies using substantially higher, non-environmental concentrations without ecotoxicological framing were noted but given less weight in formulating conclusions. Recent studies published within the last five years were prioritized, although earlier studies were also included when considered directly relevant to the review objectives. Final article selection was based on title, abstract, and full-text screening.

## Results and discussion

### 3.1. General dataset information

Table 1 presents an overview of the bibliometric dataset analysed in this study. A total of 1,051 articles published between 2010 and 2025 were retrieved from the Scopus database, originating from 175 scientific journals and involving 4,402 authors. The average number of citations was 47.1 per article, with an annual publication growth rate of 6.68%. Overall, these articles cited 6,951 references and included 1,956 author keywords.

**Table 1.** Summary of publication data used in the bibliometric analysis between 2010 and 2025.

Description	Results
MAIN INFORMATION ABOUT DATA	
Timespan	2010:2025
Sources (Journals, Books, etc)	175
Documents	1051
Annual growth rate %	6.68
Document average age (years)	6.45
Average citations per document	47.1
References	6951
DOCUMENT CONTENTS	
Keywords Plus (ID)	10559
Author’s keywords (DE)	1956
AUTHORS	
Authors	4402
Authors of single-authored documents	0
AUTHORS COLLABORATION	
Single-authored documents	0
Co-authors per document	11.7
International co-authorships %	32.92
DOCUMENT TYPES	
Journal Article	1051

### 3.2. Publication trends

**Figure 2.**
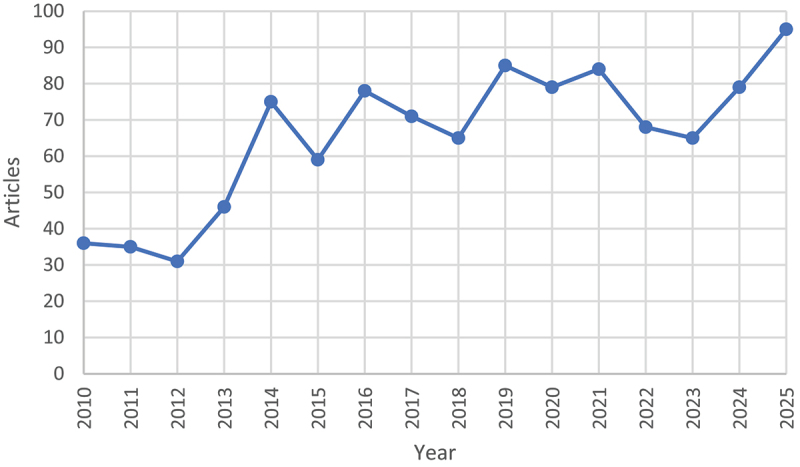
Trend in the number of annual publications related to pharmaceutical waste in ecotoxicology studies during the period 2010–2025.

The development of research on pharmaceutical waste in ecotoxicology is reflected in changes in the number of annual publications during the period from 2010 to 2025 (Figure 2). Although publication output was relatively limited during the early years, research activity increased substantially over time and remained consistently high in recent years. Overall, this pattern aligns with the annual publication growth rate of 6.68%, indicating growing scientific attention towards pharmaceutical waste as an emerging environmental concern in ecotoxicology studies.

The increasing publication trend may be associated with greater awareness of the chronic biological effects of pharmaceutical contaminants, even at environmentally relevant concentrations [24]. In addition, advances in analytical detection technologies and environmental monitoring strategies have facilitated the identification of pharmaceutical residues in wastewater and aquatic ecosystems, thereby contributing to the expansion of research activity in this field [25].

### 3.3. Country contributions

**Figure 3.**
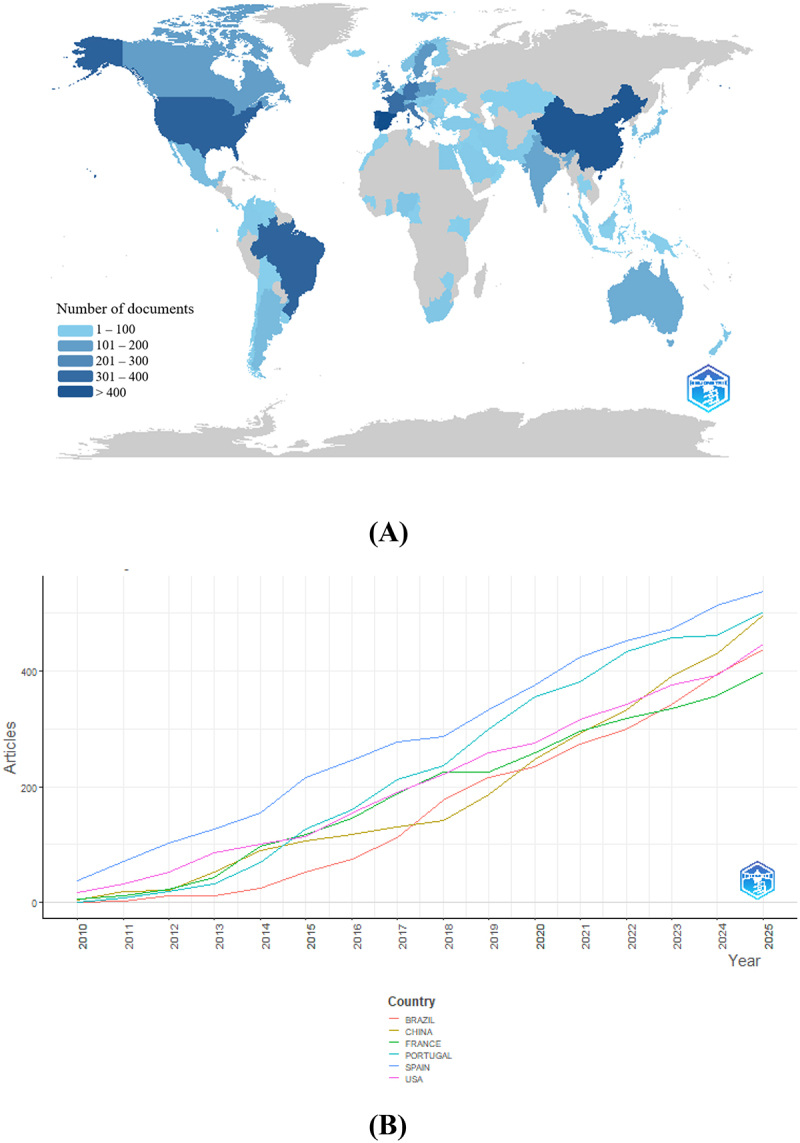
Country contributions to pharmaceutical waste research in ecotoxicology. (A) Geographic distribution map of publications based on the number of documents per country, where darker shades indicate a higher number of publications. (B) Trend in the annual number of publications of the highest-contributing countries during the 2010–2025 period.

Country scientific production was analysed in Biblioshiny using author affiliation data extracted from bibliographic records. The analysis was based on author appearances by country affiliations, in which each publication was attributed to all countries represented by the affiliations of contributing authors [26]. Overall, 79 countries contributed to pharmaceutical waste research in ecotoxicology during the 2010–2025 period (Figure 3). The publication distribution map (Figure 3(A)) shows that darker shades represent a higher number of publications. Based on this visualization, the five countries with the highest contributions are Spain (*n* = 537), Portugal (*n* = 502), China (*n* = 496), the United States (*n* = 446), and Brazil (*n* = 437).

The annual publication trends of the highest-contributing countries are shown in Figure 3(B). Spain and Portugal exhibited a relatively stable pattern of increasing publications throughout the observation period, reflecting a consistent research focus on pharmaceutical waste as a key issue in ecotoxicology, particularly in aquatic systems [27,28]. Meanwhile, China, the United States, and Brazil showed a more gradual pattern of increase, reflecting the expanding global attention to pharmaceutical waste in ecotoxicology [29–31]. The predominance of Spain and Portugal may be associated with the strong research emphasis on environmental pollution, wastewater management, and aquatic ecotoxicology within European scientific networks. European countries have been actively involved in monitoring emerging contaminants, including pharmaceutical residues, particularly in wastewater and aquatic environments, which may have contributed to the high research output observed in this field [32].

### 3.4. Top journals

**Figure 4.**
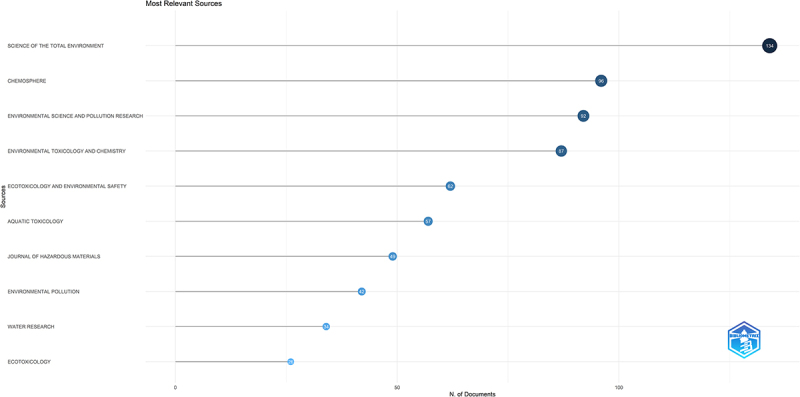
Distribution of the top 10 journals based on the number of publications related to pharmaceutical waste in ecotoxicology studies during the period 2010–2025.

Overall, 175 scientific journals contributed publications related to pharmaceutical waste and ecotoxicology during the period 2010–2025. To identify the main publication sources in this field, further analysis focused on the top 10 journals based on the number of articles published. The distribution of journal productivity is shown in Figure 4, while a breakdown of the number of publications (n) and total citations (TC) is presented in Table 2.

**Table 2.** Top 10 journals contributing publications related to pharmaceutical waste and ecotoxicology during the period 2010–2025.

Sources	Documents	Total Citations
SCIENCE OF THE TOTAL ENVIRONMENT	134	8941
CHEMOSPHERE	96	4843
ENVIRONMENTAL SCIENCE AND POLLUTION RESEARCH	92	3619
ENVIRONMENTAL TOXICOLOGY AND CHEMISTRY	87	2491
ECOTOXICOLOGY AND ENVIRONMENTAL SAFETY	62	2854
AQUATIC TOXICOLOGY	57	3463
JOURNAL OF HAZARDOUS MATERIALS	49	3300
ENVIRONMENTAL POLLUTION	42	2106
WATER RESEARCH	34	3904
ECOTOXICOLOGY	26	871

The analysis identified *Science of the Total Environment*, *Chemosphere*, and *Environmental Science and Pollution Research* as the leading publication sources in this field, based on publication output and citation impact. Other influential journals included *Environmental Toxicology and Chemistry*, *Ecotoxicology and Environmental Safety*, *Aquatic Toxicology*, and *Journal of Hazardous Materials*. These journals predominantly focus on environmental pollution and aquatic toxicology, indicating that pharmaceutical ecotoxicology research has largely developed within water-based environmental frameworks.

### 3.5. Reference co-citation analysis

**Figure 5.**
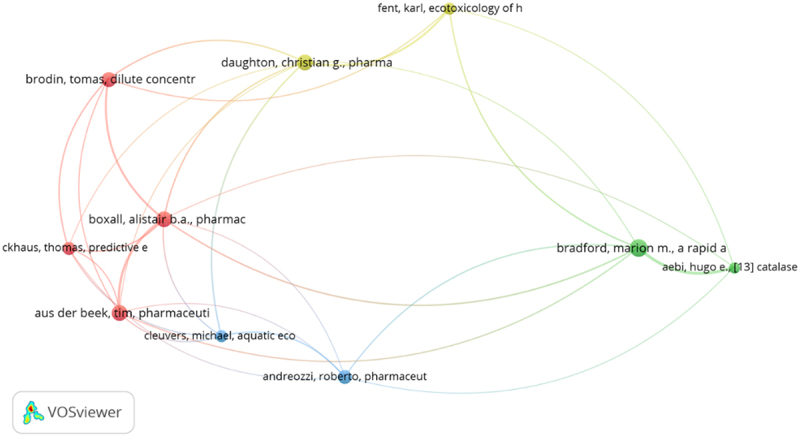
Reference co-citation network of highly cited studies in pharmaceutical waste and ecotoxicology research during 2010–2025. Node size represents co-citation frequency, while links indicate co-citation relationships between references. Colors represent clusters of related studies.

Reference co-citation analysis was performed to identify influential studies that form the intellectual foundation of pharmaceutical waste research in ecotoxicology. The analysis generated a network of highly cited references grouped into several clusters (Figure 5), indicating the presence of different thematic areas within the literature.

Several references occupy central positions in the network, particularly studies by Boxall et al. Aus der Beek et al. Daughton et al. Bradford et al. Andreozzi et al. and Brodin et al. which are frequently co-cited in the literature and mainly address the occurrence, environmental distribution, and ecological risks of pharmaceutical contaminants in the environment. Studies occupying central nodes are predominantly concerned with environmental biology, hydrology, and the regulatory dimensions of pharmaceutical contaminants.

### 3.6. Most influential authors

Analysis of authorship in research related to pharmaceutical waste and ecotoxicology revealed the involvement of 4,402 authors during the period 2010–2025. The top ten authors, as presented in Table 3, demonstrate outstanding contributions in terms of both productivity and citation rates. These authors generally contribute to studies on the occurrence and impacts of pharmaceutical waste in the environment, including research on toxicity and bioaccumulation in both aquatic and non-aquatic organisms [33–37].

**Table 3.** Most productive and highly cited authors in studies on the ecotoxicology of pharmaceutical waste.

Authors	Documents	Citations
NUNES, B. ANDRÉ FERNANDES DE JESUS DA SILVA	29	1236
BARCELÓ, DAMIÀ À.	22	2094
SOARES, AMADEU M.V.M.	20	733
BROOKS, BRYAN W.	16	767
WONG, BOB B.M.	15	439
BRODIN, TOMAS	14	475
FICK, JERKER BERGLUND	13	637
OWEN, STEWART F.	13	460
SUMPTER, JOHN P.	13	1153
ANTUNES, S. C.	12	687

Several authors with a high number of publications and citation counts, such as Damià A. Barceló (*n* = 22; TC = 2,094) and John P. Sumpter (*n* = 13; TC = 1,153), are closely associated with research on pharmaceutical contaminants as emerging pollutants, including studies addressing the long-term effects of low-dose exposure [38–41]. Meanwhile, authors such as B. André Fernandes de Jesus da Silva Nunes (*n* = 29; TC = 1,236) and Amadeu M.V.M. Soares (*n* = 20; TC = 733) frequently engage in experimental studies evaluating the physiological and molecular responses of organisms to exposure to pharmaceutical compounds and their mixtures [42–44]. This diversity of research approaches reflects the broad scope of pharmaceutical ecotoxicology that has developed over the last two decades, and the disciplinary breadth underscores the requirement of multidisciplinary collaboration across these fields.

### Keyword analysis

**Figure 6.**
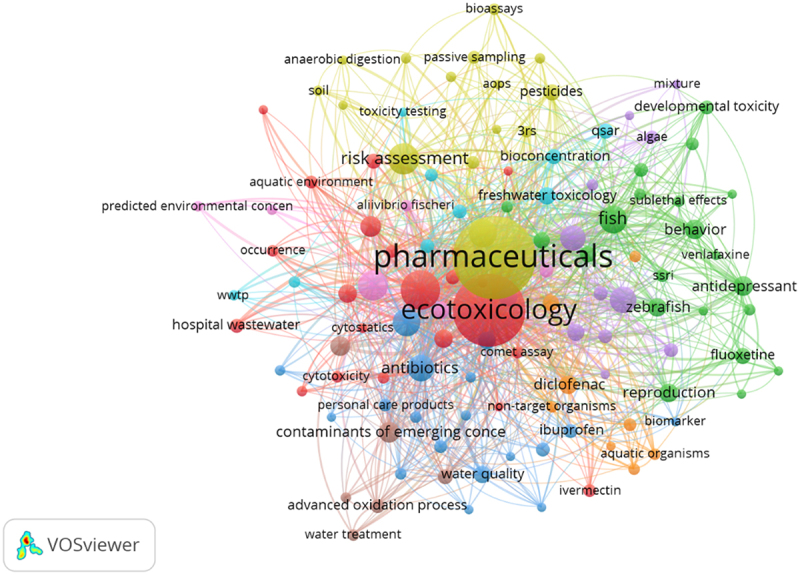
Map of keyword co-occurrence in pharmaceutical waste research in ecotoxicology studies (2010–2025).

Keyword co-occurrence analysis was performed to identify dominant research themes, conceptual relationships, and emerging directions in pharmaceutical ecotoxicology studies [45,46]. The co-occurrence network was generated using VOSviewer based on author keywords extracted from the analysed documents. A minimum occurrence threshold of 7 was applied to a total of 1,956 keywords, resulting in 118 keywords that met the criteria for visualization and network analysis (Figure 6) [46,47]. The size of each node represents keyword occurrence frequency, whereas the distance and connecting lines indicate the strength of conceptual relationships among terms within the network.

Table 4 lists the top keywords ranked according to occurrence frequency and total link strength. The most frequent keywords were *pharmaceuticals*, *ecotoxicology*, *environmental risk*, *risk assessment*, and *wastewater*, with 466, 354, 106, 67, and 61 occurrences and total link strengths of 1008, 770, 250, 157, and 165, respectively. Other highly associated keywords included *emerging contaminants*, *fish*, *biomarkers*, *toxicity*, and *antibiotics*. In addition, terms such as *oxidative stress*, *surface water*, and *zebrafish* were also observed within the co-occurrence network. Collectively, these patterns indicate that pharmaceutical ecotoxicology research is strongly associated with contaminant occurrence, environmental risk assessment, and toxicological evaluation in aquatic environments [48–52]. This pattern also reflects the broad scope of the bibliometric dataset, which includes studies addressing environmental occurrence, chemical monitoring, risk assessment, and biological effects of pharmaceutical contaminants across different environmental systems.

**Table 4.** Most frequently used keywords ranked by total link strength.

Keyword	Occurrences	Total link strength
Pharmaceuticals	466	1008
Ecotoxicology	354	770
Environmental risk	106	250
Wastewater	61	165
Risk assessment	67	157
Emerging contaminants	55	148
Fish	53	128
Biomarkers	47	117
Toxicity	55	111
Antibiotics	49	110

Several terms related to terrestrial contexts and environmental transformation processes, including *soil*, *transformation products*, and *anaerobic digestion*, were also identified within the network, although with lower occurrences and weaker link strengths compared with aquatic-related terms. These findings indicate that terrestrial environmental pathways are present but remain less developed within the current pharmaceutical ecotoxicology landscape, while aquatic-oriented approaches continue to dominate the current bibliometric structure.

### 3.8. Insect-based pharmaceutical ecotoxicology

The bibliometric patterns identified in this study suggest that pharmaceutical ecotoxicology research has largely developed within aquatic environmental frameworks, while terrestrial exposure systems remain comparatively less emphasized. This distribution highlights the need for further exploration of contaminant transfer processes occurring within terrestrial ecosystems and food webs.

In this context, insects represent ecologically relevant organisms because of their involvement in decomposition, waste conversion, and trophic interactions that may contribute to contaminant transfer in terrestrial environments. Based on this bibliometric evidence, the present narrative review subsequently focuses on two insect models with complementary ecological and mechanistic relevance. *H. illucens* was selected because of its established role in organic waste bioconversion and contaminant processing [12,53], whereas *D. melanogaster* was selected due to its extensive application as a mechanistic model for evaluating physiological and molecular responses to pharmaceutical exposure [15,54,55]. Accordingly, the following sections extend the bibliometric findings into a narrative discussion of insect-mediated approaches in terrestrial pharmaceutical ecotoxicology.

Nevertheless, it is important to note that other terrestrial models, such as *Eisenia fetida* (an OECD standard for soil toxicity) and bees (*Apis mellifera*), are also highly relevant to pharmaceutical ecotoxicology and should be considered alongside the insect models proposed here in a fully integrated framework.

### 3.9. Pharmaceutical waste in the food chain

Pharmaceutical waste has been identified as an emerging contaminant that is frequently detected in the environment and has the potential to enter the food chain [6,56,57]. Persistent pharmaceutical compounds can accumulate in organisms at lower trophic levels through exposure to contaminated water, sediment, or food sources, including invertebrates and insects [58,59]. This process allows for initial bioaccumulation, which underlies the transfer of biologically active compounds within trophic networks.

Once accumulated in prey, pharmaceutical residues do not stop; ingestion by predators completes a trophic relay that can sustain and even amplify biological exposure well beyond what single-organism assays would suggest [60–63]. This fact is consequential at environmentally relevant, chronically low concentrations, where effects may be subtle but cumulative across generations. Thus, understanding the dynamics of bioaccumulation and transfer is crucial for evaluating the long-term implications of pharmaceutical waste exposure across different trophic levels.

### 3.10. Toxic effects on organisms and biological systems

Unlike conventional pollutants, pharmaceuticals are by design biologically active at trace concentrations, a property that makes them uniquely problematic for non-target organisms, including more complex biological systems, which encounter them without the evolutionary pressure to tolerate their specific mechanisms of action [64–67]. At the physiological level, pharmaceutical compounds can affect reproduction, growth, and development, and induce oxidative stress characterized by increased production of reactive oxygen species and alterations in antioxidant defence systems [68–70]. These effects are often observed even at relatively low environmental concentrations, raising concerns about long-term chronic impacts.

In addition to physiological effects, pharmaceutical waste can also induce systemic disruptions, including alterations in metabolic regulation, hormonal signalling, and immune responses [71,72]. Several classes of pharmaceutical compounds, such as antibiotics, analgesics, and endocrine-active drugs, have been reported to disrupt energy homoeostasis, endocrine function, and immune activity in exposed organisms [73–77]. These findings indicate that the impacts of pharmaceutical waste are not limited to localized or acute effects but may also influence biological functions in an integrated manner, highlighting the importance of evaluating these effects using model organisms capable of capturing systemic responses.

### 3.11. *Insects as dual-role models in trophic exposure studies:* Hermetia illucens *and* Drosophila melanogaster

**Figure 7.**
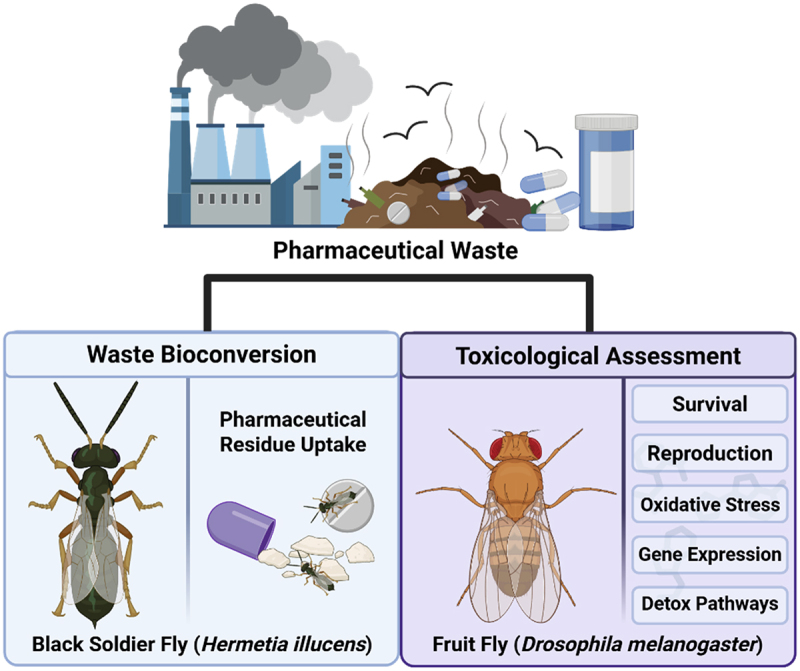
Roles of *Hermetia illucens* and *Drosophila melanogaster* in pharmaceutical ecotoxicology.

The framing of insect involvement in pharmaceutical ecotoxicology has lagged behind empirical necessity, a gap best illustrated by examining two fly species whose contrasting ecological roles make them indispensable (Figure 7). One species frequently reported is *H. illucens*, which plays a key role in the management and bioconversion of organic waste [78–80]. Previous studies have shown that *H. illucens* is capable of processing waste containing residues of pharmaceutical compounds, potentially reducing pollutant loads through biological mechanisms [12,81]. With this capacity, *H. illucens* represents a relevant candidate for exploration as an insect-based approach to pharmaceutical waste management, particularly at upstream stages before pollutants enter broader ecological systems.

In contrast, *D. melanogaster* is a well-established model organism in genetics and toxicology and is widely used to evaluate the physiological, biochemical, and molecular impacts of chemical compound exposure [82–84]. Pharmaceutical compound exposure in *Drosophila* has been reported to affect various biological parameters, including survival, reproduction, oxidative stress, and the regulation of gene expression related to metabolism and stress responses [85–88]. The high degree of conservation of biological pathways and drug targets, with approximately 75% of human disease-related genes conserved, combined with the ease of experimental manipulation and the extensive genetic toolkit available (e.g. GAL4/UAS, CRISPR), makes *D. melanogaster* an effective toxicological sentinel for assessing systemic impacts of pharmaceutical waste exposure, including chronic low-dose exposures that may be relevant to organisms at higher trophic levels.

Beyond its value as a toxicological model, *D. melanogaster* also provides mechanistic insights into the biological effects of pharmaceutical exposure. For instance, exposure to acetaminophen (paracetamol) has been reported to alter life-history traits such as fecundity, lifespan, and locomotor activity, with effects that may persist across generations [89]. In addition, experimental models have demonstrated that acetaminophen exposure can induce oxidative stress through increased production of reactive oxygen species (ROS) and depletion of antioxidant defences [90]. Exposure to other pharmaceutical compounds, such as fluoxetine, a selective serotonin reuptake inhibitor (SSRI), has also been reported to affect the serotonergic signalling pathway in *Drosophila*, which plays an important role in regulating behaviour and physiological functions [91].

The relevance of *D. melanogaster* as a toxicological model extends to the conservation of the underlying molecular machinery. Many detoxification pathways in *D. melanogaster* are evolutionarily conserved with those of higher organisms, including cytochrome P450 (CYP450) enzymes that play a key role in xenobiotic metabolism and the biotransformation of pharmaceutical compounds. Insecticide resistance in *Drosophila* demonstrates that constitutive activation of the Nrf2/CncC pathway can upregulate CYP450 genes [92]. CncC-mediated redox signalling helps fight oxidative insults and regulates redox balance, including due to pollutant [93,94]. Furthermore, the well-characterized nervous system of *Drosophila* allows the assessment of neurobehavioral endpoints, including locomotor activity, climbing ability, and feeding behaviour, which serve as sensitive indicators of neurotoxicity and systemic physiological disruption following chemical exposure [95].

Conceptually, the combined use of *H. illucens* and *D. melanogaster* represents a continuum of roles in pharmaceutical ecotoxicology. *H. illucens* functions primarily in the management and reduction of pharmaceutical waste at the environmental level, while *Drosophila* enables further evaluation of the biological and molecular consequences of exposure to these compounds. This insect-based approach has the potential to address limitations associated with conventional models, which have been largely dominated by aquatic organisms, and to open new opportunities for developing a more integrative, cross-trophic, and ecologically relevant framework for pharmaceutical ecotoxicology that better reflects pollution dynamics in real-world environments.

## Conclusion

Bibliometric analysis indicates that research on pharmaceutical waste in ecotoxicology has continued to increase globally during the period 2010–2025, with a predominance of publications focusing on aquatic environments and aquatic organisms. Keyword patterns and research themes indicate that studies examining exposure pathways through terrestrial food chains remain relatively limited.

The narrative review reveals that pharmaceutical waste can accumulate in organisms and be transferred across trophic levels, leading to a range of physiological and systemic toxic effects, including oxidative stress and disruption of biological regulation, even at low environmental concentrations. However, most of the available evidence continues to originate from aquatic systems.

Based on these findings, insects have the potential to serve as valuable non-aquatic models for studying trophic exposure to pharmaceutical waste, and we propose an insect-based ecotoxicology framework in which *H. illucens* acts as a bioconversion model for waste processing and *D. melanogaster* as a mechanistic toxicological model. Fly-based approaches, already trusted in mechanistic studies across genetics, ageing, and neuroscience, stand ready to assume an equivalent role in ecotoxicology and help broaden our understanding of pharmaceutical waste exposure dynamics. For that, deliberate multidisciplinary collaboration, integrating ecology, entomology, molecular biology, environmental toxicology, and waste management, will be essential to more fully account for the trophic and terrestrial dimensions of the problem.

## Data Availability

All data generated or analysed during this study are included in this manuscript.
